# 2-Methyl-*N*-(4-methyl­phen­yl)benzamide

**DOI:** 10.1107/S1600536808021235

**Published:** 2008-07-16

**Authors:** B. Thimme Gowda, Miroslav Tokarčík, Jozef Kožíšek, B. P. Sowmya, Hartmut Fuess

**Affiliations:** a Department of Chemistry, Mangalore University, Mangalagangotri 574 199, Mangalore, India; b Faculty of Chemical and Food Technology, Slovak Technical University, Radlinského 9, SK-812 37 Bratislava, Slovak Republic; c Institute of Materials Science, Darmstadt University of Technology, Petersenstrasse 23, D-64287 Darmstadt, Germany

## Abstract

The conformations of the N—H and C=O bonds in the structure of the title compound, C_15_H_15_NO, are *trans* to each other. Furthermore, the position of the amide O atom is *syn* to the *ortho*-methyl group in the benzoyl ring. The central amide group is tilted at an angle of 59.96 (11)° to the benzoyl ring, and the benzoyl and aniline rings form a dihedral angle of 81.44 (5)°. N—H⋯O hydrogen bonds link the mol­ecules into infinite chains running along the *c* axis.

## Related literature

For related literature, see Gowda *et al.* (2003 ▶, 2008**a* ▶,b* ▶,*c* ▶).
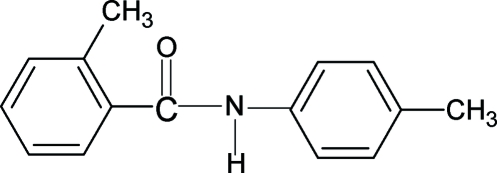


## Experimental

### 

#### Crystal data

C_15_H_15_NO
*M* *_r_* = 225.28
Monoclinic, 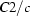

*a* = 40.6634 (12) Å
*b* = 7.1770 (2) Å
*c* = 8.9418 (2) Å
β = 96.173 (3)°
*V* = 2594.45 (12) Å^3^
*Z* = 8
Mo *K*α radiation
μ = 0.07 mm^−1^
*T* = 295 (2) K
0.33 × 0.13 × 0.10 mm

#### Data collection

Oxford Diffraction Xcalibur System diffractometer
Absorption correction: multi-scan (*CrysAlis RED*; Oxford Diffraction, 2007 ▶) *T*_min_ = 0.985, *T*_max_ = 0.994
25372 measured reflections
2486 independent reflections
1594 reflections with *I* > 2σ(*I*)
*R*_int_ = 0.049

#### Refinement

*R*[*F* ^2^ > 2σ(*F* ^2^)] = 0.045
*wR*(*F* ^2^) = 0.121
*S* = 1.02
2486 reflections
159 parameters
4 restraints
H atoms treated by a mixture of independent and constrained refinement
Δρ_max_ = 0.13 e Å^−3^
Δρ_min_ = −0.11 e Å^−3^

### 

Data collection: *CrysAlis CCD* (Oxford Diffraction, 2007 ▶); cell refinement: *CrysAlis RED* (Oxford Diffraction, 2007 ▶); data reduction: *CrysAlis RED*; program(s) used to solve structure: *SHELXS97* (Sheldrick, 2008 ▶); program(s) used to refine structure: *SHELXL97* (Sheldrick, 2008 ▶); molecular graphics: *ORTEP-3* (Farrugia, 1997 ▶) and *DIAMOND* (Brandenburg, 2002 ▶); software used to prepare material for publication: *SHELXL97*, *PLATON* (Spek, 2003 ▶) and *WinGX* (Farrugia, 1999 ▶).

## Supplementary Material

Crystal structure: contains datablocks I, global. DOI: 10.1107/S1600536808021235/tk2283sup1.cif

Structure factors: contains datablocks I. DOI: 10.1107/S1600536808021235/tk2283Isup2.hkl

Additional supplementary materials:  crystallographic information; 3D view; checkCIF report

## Figures and Tables

**Table 1:** Hydrogen-bond geometry (Å, °)

*D*—H⋯*A*	*D*—H	H⋯*A*	*D*⋯*A*	*D*—H⋯*A*
N1—H1*N*⋯O1^i^	0.868 (13)	1.973 (13)	2.8361 (15)	172.9 (15)

Symmetry code: (i) 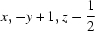.

**Fig. 1.**
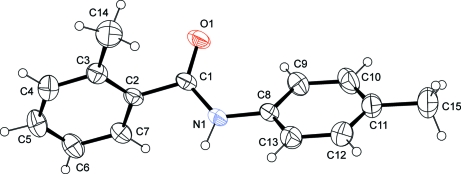
Molecular structure of (I) showing the atom labelling scheme and displacement ellipsoids at the 30% probability level.

**Fig. 2.**
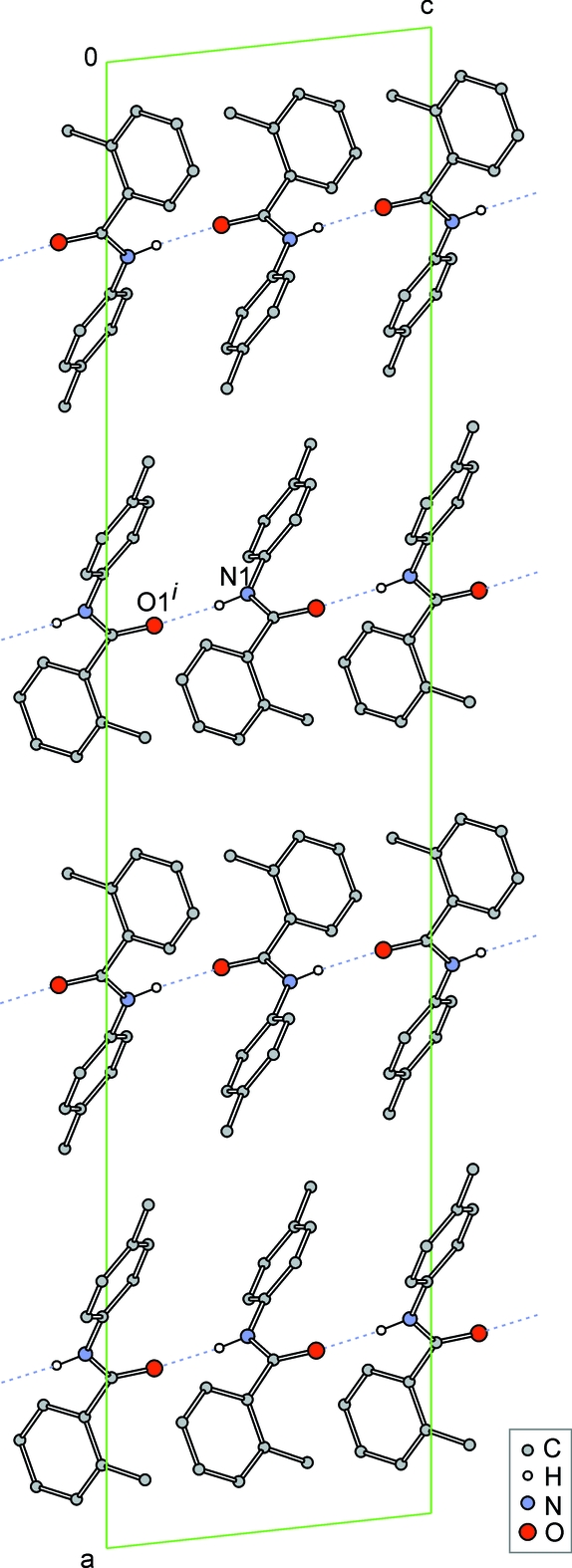
Part of crystal structure of (I) viewed down the b-axis. Hydrogen bonds N1–H1N···O1(i) give rise to infinite molecular chains running along the c axis. Symmetry code: (i) x,-y + 1,z - 1/2. H atoms not involved in hydrogen bonding are omitted and hydrogen bonds are shown as dashed lines.

## supplementary crystallographic information

### Comment

As part of a study of exploring the effect of substituents on the structures of benzanilides (Gowda *et al.*, 2003; 2008*a*,*b*,*c*), inthe present work, the structure of 2-methyl-*N*-(4-methylphenyl)benzamide (I) has been determined. The N—H and C=O bonds in the amide group of (I) are *trans* to each other (Fig. 1), similar to what is observed in *N*-(4-methylphenyl)benzamide (N4MPBA) (Gowda *et al.*, 2008*c*), 2-methyl-*N*-(phenyl)-benzamide (NP2MBA) (Gowda *et al.*, 2008*a*), and 2-methyl-*N*-(3-methylphenyl)-benzamide (N3MP2MBA) (Gowda *et al.*, 2008*b*). Further, the conformation of the amide oxygen in (I) is *syn* to the *ortho*-methyl group in the benzoyl ring, similar to what is observed in NP2MBA and N3MP2MBA (Gowda *et al.*, 2008*a, b*).

The central amide group is tilted to the benzoyl ring at the angle of 59.96 (11)°. The two rings (benzoyl and aniline) make a dihedral angle of 81.44 (5)°. N—H···O hydrogen bonds link the molecules into infinite chains running along the *c*-axis of the crystal (Table 1 & Fig. 2).

### Experimental

The title compound was prepared according to the method described by Gowda *et al.* (2003). Prism-like colourless single crystals were obtained by slow evaporation from an ethanol solution of (I) (0.5 g in about 40 ml of ethanol) at room temperature.

### Refinement

All C-bound H atoms were placed in calculated positions and constrained to ride on their parent atoms with C–H = 0.93- 0.96 Å. The H atoms of C15-methyl group were finally refined as orientationally disordered using the AFIX 127 command in SHELXL-97 (Sheldrick, 2008). The amide-H atom was refined with the N–H distance restrained to 0.86 (2) Å. The *U*_iso_(H) values were set at 1.2*U*_eq_(C,*N*) and 1.5*U*_eq_ (*C*-methyl). The displacement parameters of three C-atoms in aniline ring were restrained using the DELU command with standard deviation of 0.003.

### Crystal data

**Table:**

C_15_H_15_NO	*F*_000_ = 960
*M**_r_* = 225.28	*D*_x_ = 1.153 Mg m^−^^3^
Monoclinic, *C*2/*c*	Mo *K*α radiation λ = 0.71073 Å
Hall symbol: -C 2yc	Cell parameters from 6446 reflections
*a* = 40.6634 (12) Å	θ = 3.0–29.2º
*b* = 7.1770 (2) Å	µ = 0.07 mm^−^^1^
*c* = 8.9418 (2) Å	*T* = 295 (2) K
β = 96.173 (3)º	Prism, colourless
*V* = 2594.45 (12) Å^3^	0.33 × 0.13 × 0.10 mm
*Z* = 8	

### Data collection

**Table:**

Oxford Diffraction Xcalibur System diffractometer	2486 independent reflections
Monochromator: graphite	1594 reflections with *I* > 2σ(*I*)
Detector resolution: 10.434 pixels mm^-1^	*R*_int_ = 0.049
*T* = 295(2) K	θ_max_ = 25.9º
ω scans with κ offsets	θ_min_ = 5.4º
Absorption correction: multi-scan(CrysAlis RED; Oxford Diffraction, 2007)	*h* = −49→49
*T*_min_ = 0.985, *T*_max_ = 0.994	*k* = −8→8
25372 measured reflections	*l* = −10→10

### Refinement

**Table:**

Refinement on *F*^2^	Secondary atom site location: difference Fourier map
Least-squares matrix: full	Hydrogen site location: inferred from neighbouring sites
*R*[*F*^2^ > 2σ(*F*^2^)] = 0.045	H atoms treated by a mixture of independent and constrained refinement
*wR*(*F*^2^) = 0.121	*w* = 1/[σ^2^(*F*_o_^2^) + (0.0661*P*)^2^ + 0.0615*P*] where *P* = (*F*_o_^2^ + 2*F*_c_^2^)/3
*S* = 1.02	(Δ/σ)_max_ = 0.001
2486 reflections	Δρ_max_ = 0.13 e Å^−^^3^
159 parameters	Δρ_min_ = −0.11 e Å^−^^3^
4 restraints	Extinction correction: none
Primary atom site location: structure-invariant direct methods	

### Special details

**Table:**

Geometry. All e.s.d.'s (except the e.s.d. in the dihedral angle between two l.s. planes) are estimated using the full covariance matrix. The cell e.s.d.'s are taken into account individually in the estimation of e.s.d.'s in distances, angles and torsion angles; correlations between e.s.d.'s in cell parameters are only used when they are defined by crystal symmetry. An approximate (isotropic) treatment of cell e.s.d.'s is used for estimating e.s.d.'s involving l.s. planes.
Refinement. Refinement of *F*^2^ against ALL reflections. The weighted *R*-factor *wR* and goodness of fit *S* are based on *F*^2^, conventional *R*-factors *R* are based on *F*, with *F* set to zero for negative *F*^2^. The threshold expression of *F*^2^ > σ(*F*^2^) is used only for calculating *R*-factors(gt) *etc*. and is not relevant to the choice of reflections for refinement. *R*-factors based on *F*^2^ are statistically about twice as large as those based on *F*, and *R*- factors based on ALL data will be even larger.

### Fractional atomic coordinates and isotropic or equivalent isotropic displacement parameters (Å^2^)

**Table:**

	*x*	*y*	*z*	*U*_iso_*/*U*_eq_	Occ. (<1)
C1	0.38556 (4)	0.3947 (2)	0.51508 (15)	0.0490 (4)	
C2	0.41020 (4)	0.29352 (18)	0.43275 (14)	0.0460 (4)	
C3	0.44366 (4)	0.2966 (2)	0.48505 (16)	0.0556 (4)	
C4	0.46488 (4)	0.1922 (2)	0.4075 (2)	0.0683 (5)	
H4	0.4874	0.193	0.4397	0.082*	
C5	0.45356 (5)	0.0877 (2)	0.2846 (2)	0.0744 (5)	
H5	0.4683	0.0168	0.236	0.089*	
C6	0.42084 (5)	0.0867 (2)	0.23294 (19)	0.0712 (5)	
H6	0.4132	0.0166	0.1488	0.085*	
C7	0.39921 (4)	0.1908 (2)	0.30672 (15)	0.0577 (4)	
H7	0.3769	0.192	0.2712	0.069*	
C8	0.34387 (4)	0.6436 (2)	0.48652 (14)	0.0508 (4)	
C9	0.32161 (4)	0.5851 (2)	0.58239 (17)	0.0654 (5)	
H9	0.3219	0.4626	0.6163	0.078*	
C10	0.29882 (4)	0.7110 (3)	0.6277 (2)	0.0785 (5)	
H10	0.2839	0.6706	0.6926	0.094*	
C11	0.29747 (4)	0.8930 (3)	0.5804 (2)	0.0752 (5)	
C12	0.32004 (5)	0.9467 (3)	0.4855 (2)	0.0799 (5)	
H12	0.3199	1.0694	0.452	0.096*	
C13	0.34283 (4)	0.8252 (3)	0.43868 (18)	0.0697 (5)	
H13	0.3577	0.8664	0.3739	0.084*	
C14	0.45688 (5)	0.4126 (3)	0.6189 (2)	0.0896 (6)	
H14A	0.4806	0.4132	0.6273	0.134*	
H14B	0.4496	0.3606	0.7087	0.134*	
H14C	0.4488	0.538	0.606	0.134*	
C15	0.27185 (5)	1.0262 (4)	0.6292 (3)	0.1144 (9)	
H15A	0.2567	0.9594	0.6848	0.172*	0.5
H15B	0.2599	1.0821	0.542	0.172*	0.5
H15C	0.2826	1.1217	0.6917	0.172*	0.5
H15D	0.2761	1.1494	0.5942	0.172*	0.5
H15E	0.2729	1.0267	0.737	0.172*	0.5
H15F	0.2502	0.9871	0.5873	0.172*	0.5
N1	0.36770 (3)	0.52332 (18)	0.43453 (13)	0.0545 (4)	
H1N	0.3735 (4)	0.552 (2)	0.3467 (15)	0.065*	
O1	0.38239 (3)	0.35884 (16)	0.64635 (11)	0.0718 (4)	

### Atomic displacement parameters (Å^2^)

**Table:**

	*U*^11^	*U*^22^	*U*^33^	*U*^12^	*U*^13^	*U*^23^
C1	0.0637 (9)	0.0501 (9)	0.0351 (7)	−0.0025 (7)	0.0132 (6)	−0.0003 (6)
C2	0.0611 (9)	0.0411 (8)	0.0374 (7)	0.0016 (7)	0.0133 (6)	0.0030 (6)
C3	0.0646 (10)	0.0491 (9)	0.0537 (9)	0.0001 (8)	0.0091 (7)	−0.0005 (7)
C4	0.0587 (10)	0.0634 (11)	0.0843 (12)	0.0030 (9)	0.0147 (9)	0.0005 (9)
C5	0.0779 (13)	0.0625 (12)	0.0871 (12)	0.0077 (10)	0.0293 (10)	−0.0164 (10)
C6	0.0846 (13)	0.0660 (12)	0.0653 (10)	−0.0002 (10)	0.0184 (9)	−0.0243 (9)
C7	0.0645 (10)	0.0597 (10)	0.0500 (8)	−0.0019 (8)	0.0107 (7)	−0.0079 (7)
C8	0.0518 (9)	0.0636 (10)	0.0376 (7)	0.0047 (8)	0.0082 (6)	−0.0023 (7)
C9	0.0613 (10)	0.0722 (11)	0.0655 (9)	−0.0040 (8)	0.0205 (8)	−0.0039 (8)
C10	0.0576 (10)	0.1063 (15)	0.0759 (11)	−0.0022 (10)	0.0265 (8)	−0.0135 (10)
C11	0.0625 (11)	0.0948 (13)	0.0667 (11)	0.0212 (10)	−0.0003 (9)	−0.0204 (10)
C12	0.0887 (13)	0.0752 (13)	0.0761 (11)	0.0250 (11)	0.0104 (10)	0.0027 (10)
C13	0.0773 (12)	0.0708 (12)	0.0641 (10)	0.0136 (10)	0.0214 (8)	0.0106 (9)
C14	0.0823 (13)	0.0965 (15)	0.0864 (12)	−0.0020 (11)	−0.0075 (10)	−0.0278 (11)
C15	0.0884 (15)	0.146 (2)	0.1076 (15)	0.0512 (15)	0.0038 (12)	−0.0354 (15)
N1	0.0660 (8)	0.0652 (9)	0.0352 (6)	0.0115 (7)	0.0185 (6)	0.0062 (6)
O1	0.1041 (9)	0.0757 (8)	0.0394 (6)	0.0222 (7)	0.0253 (5)	0.0099 (5)

### Geometric parameters (Å, °)

**Table:**

C1—O1	1.2219 (15)	C9—H9	0.93
C1—N1	1.3362 (18)	C10—C11	1.372 (3)
C1—C2	1.4941 (19)	C10—H10	0.93
C2—C7	1.380 (2)	C11—C12	1.370 (3)
C2—C3	1.390 (2)	C11—C15	1.513 (3)
C3—C4	1.384 (2)	C12—C13	1.371 (2)
C3—C14	1.509 (2)	C12—H12	0.93
C4—C5	1.369 (2)	C13—H13	0.93
C4—H4	0.93	C14—H14A	0.96
C5—C6	1.361 (2)	C14—H14B	0.96
C5—H5	0.93	C14—H14C	0.96
C6—C7	1.375 (2)	C15—H15A	0.96
C6—H6	0.93	C15—H15B	0.96
C7—H7	0.93	C15—H15C	0.96
C8—C13	1.372 (2)	C15—H15D	0.96
C8—C9	1.377 (2)	C15—H15E	0.96
C8—N1	1.4132 (19)	C15—H15F	0.96
C9—C10	1.386 (2)	N1—H1N	0.868 (13)
			
O1—C1—N1	123.78 (13)	C13—C12—H12	119
O1—C1—C2	121.10 (13)	C12—C13—C8	120.57 (17)
N1—C1—C2	115.12 (11)	C12—C13—H13	119.7
C7—C2—C3	120.17 (13)	C8—C13—H13	119.7
C7—C2—C1	119.10 (13)	C3—C14—H14A	109.5
C3—C2—C1	120.69 (12)	C3—C14—H14B	109.5
C4—C3—C2	117.58 (14)	H14A—C14—H14B	109.5
C4—C3—C14	120.54 (15)	C3—C14—H14C	109.5
C2—C3—C14	121.86 (14)	H14A—C14—H14C	109.5
C5—C4—C3	121.66 (16)	H14B—C14—H14C	109.5
C5—C4—H4	119.2	C11—C15—H15A	109.5
C3—C4—H4	119.2	C11—C15—H15B	109.5
C6—C5—C4	120.51 (15)	H15A—C15—H15B	109.5
C6—C5—H5	119.7	C11—C15—H15C	109.5
C4—C5—H5	119.7	H15A—C15—H15C	109.5
C5—C6—C7	119.13 (15)	H15B—C15—H15C	109.5
C5—C6—H6	120.4	C11—C15—H15D	109.5
C7—C6—H6	120.4	H15A—C15—H15D	141.1
C6—C7—C2	120.93 (15)	H15B—C15—H15D	56.3
C6—C7—H7	119.5	H15C—C15—H15D	56.3
C2—C7—H7	119.5	C11—C15—H15E	109.5
C13—C8—C9	118.91 (15)	H15A—C15—H15E	56.3
C13—C8—N1	118.50 (14)	H15B—C15—H15E	141.1
C9—C8—N1	122.59 (15)	H15C—C15—H15E	56.3
C8—C9—C10	119.25 (17)	H15D—C15—H15E	109.5
C8—C9—H9	120.4	C11—C15—H15F	109.5
C10—C9—H9	120.4	H15A—C15—H15F	56.3
C11—C10—C9	122.42 (17)	H15B—C15—H15F	56.3
C11—C10—H10	118.8	H15C—C15—H15F	141.1
C9—C10—H10	118.8	H15D—C15—H15F	109.5
C12—C11—C10	116.86 (16)	H15E—C15—H15F	109.5
C12—C11—C15	121.9 (2)	C1—N1—C8	126.63 (12)
C10—C11—C15	121.3 (2)	C1—N1—H1N	117.8 (11)
C11—C12—C13	122.00 (18)	C8—N1—H1N	114.6 (11)
C11—C12—H12	119		
			
O1—C1—C2—C7	118.96 (16)	C13—C8—C9—C10	0.0 (2)
N1—C1—C2—C7	−60.69 (18)	N1—C8—C9—C10	−179.59 (14)
O1—C1—C2—C3	−58.66 (19)	C8—C9—C10—C11	0.1 (3)
N1—C1—C2—C3	121.68 (15)	C9—C10—C11—C12	−0.4 (3)
C7—C2—C3—C4	−0.8 (2)	C9—C10—C11—C15	178.70 (16)
C1—C2—C3—C4	176.82 (14)	C10—C11—C12—C13	0.4 (3)
C7—C2—C3—C14	177.70 (15)	C15—C11—C12—C13	−178.61 (17)
C1—C2—C3—C14	−4.7 (2)	C11—C12—C13—C8	−0.3 (3)
C2—C3—C4—C5	−0.7 (2)	C9—C8—C13—C12	0.0 (2)
C14—C3—C4—C5	−179.15 (16)	N1—C8—C13—C12	179.69 (14)
C3—C4—C5—C6	1.4 (3)	O1—C1—N1—C8	3.2 (2)
C4—C5—C6—C7	−0.6 (3)	C2—C1—N1—C8	−177.12 (14)
C5—C6—C7—C2	−0.8 (2)	C13—C8—N1—C1	139.57 (16)
C3—C2—C7—C6	1.5 (2)	C9—C8—N1—C1	−40.8 (2)
C1—C2—C7—C6	−176.10 (14)		

### Hydrogen-bond geometry (Å, °)

**Table:**

*D*—H···*A*	*D*—H	H···*A*	*D*···*A*	*D*—H···*A*
N1—H1N···O1^i^	0.868 (13)	1.973 (13)	2.8361 (15)	172.9 (15)

Symmetry codes: (i) *x*, −*y*+1, *z*−1/2.
